# The Cerebral Microvasculature in Schizophrenia: A Laser Capture Microdissection Study

**DOI:** 10.1371/journal.pone.0003964

**Published:** 2008-12-17

**Authors:** Laura W. Harris, Matthew Wayland, Martin Lan, Margaret Ryan, Thomas Giger, Helen Lockstone, Irene Wuethrich, Michael Mimmack, Lan Wang, Mark Kotter, Rachel Craddock, Sabine Bahn

**Affiliations:** 1 Department of Chemical Engineering and Biotechnology, University of Cambridge, Cambridge, United Kingdom; 2 Max Planck Institute for Evolutionary Anthropology, Leipzig, Germany; 3 Cambridge Centre for Brain Repair, Department of Veterinary Medicine, University of Cambridge, Cambridge, United Kingdom; Chiba University Center for Forensic Mental Health, Japan

## Abstract

**Background:**

Previous studies of brain and peripheral tissues in schizophrenia patients have indicated impaired energy supply to the brain. A number of studies have also demonstrated dysfunction of the microvasculature in schizophrenia patients. Together these findings are consistent with a hypothesis of blood-brain barrier dysfunction in schizophrenia. In this study, we have investigated the cerebral vascular endothelium of schizophrenia patients at the level of transcriptomics.

**Methodology/Principal Findings:**

We used laser capture microdissection to isolate both microvascular endothelial cells and neurons from post mortem brain tissue from schizophrenia patients and healthy controls. RNA was isolated from these cell populations, amplified, and analysed using two independent microarray platforms, Affymetrix HG133plus2.0 GeneChips and CodeLink Whole Human Genome arrays. In the first instance, we used the dataset to compare the neuronal and endothelial data, in order to demonstrate that the predicted differences between cell types could be detected using this methodology. We then compared neuronal and endothelial data separately between schizophrenic subjects and controls. Analysis of the endothelial samples showed differences in gene expression between schizophrenics and controls which were reproducible in a second microarray platform. Functional profiling revealed that these changes were primarily found in genes relating to inflammatory processes.

**Conclusions/Significance:**

This study provides preliminary evidence of molecular alterations of the cerebral microvasculature in schizophrenia patients, suggestive of a hypo-inflammatory state in this tissue type. Further investigation of the blood-brain barrier in schizophrenia is warranted.

## Introduction

Despite decades of research and numerous competing hypotheses, our understanding of the pathophysiology of schizophrenia remains unclear, with adverse consequences for both diagnosis and treatment. In recent years an increasing body of evidence has pointed towards altered glucose metabolism in schizophrenic patients. In addition to the major findings of hypofrontality in patients obtained using brain imaging methods [1], numerous post mortem studies have shown alterations in the expression of genes and proteins involved in major energy metabolism pathways [2]–[6], and studies of peripheral tissues have also detected metabolic alterations in first onset and drug naïve patients [7], [8]. Examination of these data suggests an abnormality in glucose utilization in the brains of patients which may arise from impaired supply of energy substrates such as glucose and lactate [5], [7]. Such findings are consistent with an hypothesis of blood-brain barrier impairment in schizophrenia [9]. This hypothesis proposes that disruption in the coupling of cerebral blood flow to neuronal metabolic needs may be upstream of all conceivable functional neuronal abnormalities in schizophrenia.

A small but growing body of evidence points towards dysfunction of the microvasculature in schizophrenia. The niacin skin flush response has been widely reported to be abnormal in schizophrenic patients [10]–[14] and may be explained by abnormal vasodilatation. Furthermore, some studies have shown a decreased resting cerebral blood flow [15], [16] and decreased cerebral vascular volume in schizophrenic patients [17], with other data suggesting an increase in blood volume in certain brain regions [18], consistent with abnormalities of the cerebral microvasculature. A recent stereological study of capillary length density in schizophrenia brain tissue failed to find differences between schizophrenic and control subjects [19], but the authors reconcile these two apparently conflicting findings by proposing dysfunction of the cerebral microvasculature at a molecular, rather than structural level. However, few if any molecular studies of the cerebral vasculature in schizophrenia have been attempted, and existing quantitative molecular studies based on tissue homogenate or sections are unlikely to include a signal from the relevant cells as vasculature accounts for only 0.1% of whole brain tissue [20].

In this study we have attempted to characterise the cerebral microvasculature of schizophrenia patients by using laser microdissection to isolate cells from post mortem prefrontal cortex tissue. Laser microdissection has been widely touted as a major advance in molecular brain research [21], but has rarely [2], [22] been applied to the human postmortem brain due to the technical challenges raised by working with small amounts of tissue and the variability that may be introduced at various stages of the analytical process. Thus as a first step to check the integrity of the data, we compared data from endothelial cells to data from neurons in order to demonstrate whether the predicted differences between these two cells types could be detected. We then proceeded to investigate gene expression differences in these cell types in schizophrenia using microarrays. Although microarray technology has been demonstrated to be robust and reproducible in careful hands, validation of the results at the technical level is essential to increase confidence in a study. Quantitative real-time PCR, the most commonly used validation tool, is limited in that only a small number of genes can practically be measured within a single study, and the necessity to normalize to a so-called “housekeeping” gene introduces high levels of experimental noise. Thus in the present study we have taken the approach of using two array platforms, each possessing different probe design, synthesis and attachment strategies and different hybridization kinetics and lab procedure. Using this approach not only can mRNA levels be validated, but also differences in microarray methodology, normalization and data processing methods.

## Methods

### Tissue collection

Consent: Human brain tissue was obtained from the Array collection of the Stanley Medical Research Institute (Bethesda, USA). Tissue was collected *post mortem* from patients and controls with full informed consent obtained from a first degree relative after death in compliance with the Declaration of Helsinki. The consent was obtained by questionnaires conducted over the phone and signed by two witnesses. All patient data are anonymised. Exemption from IRB approval was granted by the Uniformed Services University of Health Sciences IRB on the grounds that specimens were obtained via informed donation from cadaveric material in accordance with federal and state regulations, the research did not encompass genetic linkage studies, and all samples were de-identified and personal information anonymised.

Fresh-frozen gray matter tissue from dorsolateral prefrontal cortex (Brodmann area 9) of 12 schizophrenia patients and 12 matched control individuals was used in the study. The cell type analysis also included 12 subjects with bipolar disorder (see Supplementary Information S1).

### Laser capture microdissection

**Figure 1:**
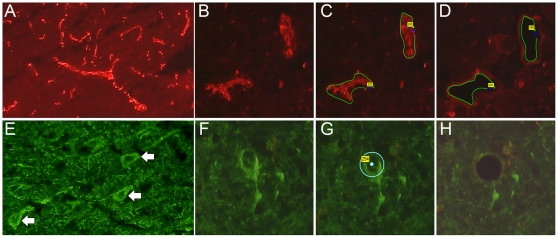
Examples of fluorescence immunostaining. A rapid immunostaining method was developed to identify endothelial cells (A–D) and neurons (E–H) in human prefrontal cortex tissue, whilst maintaining optimal RNA preservation. (A, E) low power (×10) images of cell staining in prefrontal cortex tissue; (B, F) high power (×40) image of cells before capture; (C, G) cells selected ready for capture (D, H) tissue sections post-capture.

Cells were visualised using a rapid fluorescence immunostaining method designed for optimal RNA preservation. Examples of immunostaining are shown in Fig 1. 15 µm tissue sections were cut onto polyethylene naphthalate membrane slides (Zeiss) and fixed for 10 minutes in acetone. After air drying, sections were incubated with either rabbit anti-von Willebrand factor (Chemicon) or mouse anti-neurofilament-160/200 kD (Cambridge Bioscience) for 5 minutes, followed by brief washing in RNase-free phosphate buffered saline (PBS) and incubation in secondary antibody for 5 minutes (Cy3-conjugated goat anti-rabbit IgG or Cy2-conjugated goat anti-mouse IgG (Jackson Immunoresearch). All antibodies were used at 1∶20 dilution with 1 unit/ml RNase inhibitor (GE Healthcare) in RNase-free PBS (Ambion). These incubation conditions were found to give the best staining for cell identification together with optimal RNA preservation. Following antibody incubation and brief washing in PBS, sections were then dehydrated through ethanol series and cell capture was initiated immediately. Laser capture microdissection was carried out using the PALM microlaser system [23]. 1000 neurons were captured from each subject, in two batches of 500, or an equivalent area of vascular endothelium (approximately 40 0000 µm2). Pyramidal neurons were selected based on staining and morphology. Following capture, RNA was extracted from cells using the PALM RNA extraction kit (Zeiss) and amplified through 2 rounds using the RiboAmp HS kit (Arcturus). The resulting aRNA was assessed on an Agilent Bioanalyser Nanochip to determine length of RNA transcripts in the samples. aRNA profiles with jagged curves or pronounced skews to the left, indicating degradation of the RNA, were eliminated from the analysis.

### Microarray hybridisation

Amplified RNA was converted to cDNA using Round 2 components of the RiboAmp HS kit, labelled by *in vitro* transcription in the presence of biotinylated UTP (Codelink Expression Assay kit, GE Healthcare), and purified using YM-30 columns (Microcon).

Labelled RNA was hybridised to both Affymetrix and Codelink chips according to manufacturers' recommendations. Balanced numbers of patient and control samples were included in each hybridisation batch.

### Data analysis

**Figure 2:**
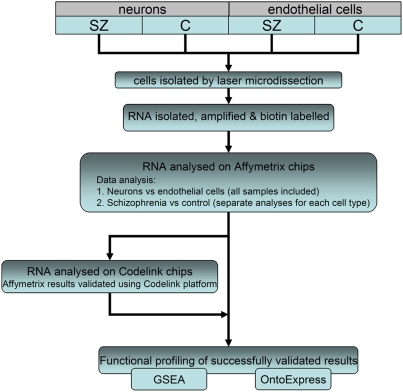
Diagram showing overview of experimental workflow.

All datasets (endothelial and neuronal, disease and control samples) were subjected to normalisation and quality control measures together, within each array platform. Only samples which passed quality control on both platforms were included in the final dataset, to facilitate cross-platform validation. An outline of the data analysis workflow is shown in Fig 2.

### Affymetrix arrays

#### Data pre-processing

Quality control protocols for Affymetrix microarray data derived from human postmortem brains were applied as previously described [24], and samples which did not pass were removed from the dataset. Expression measures were computed for each of the probesets on each of the GeneChips in the dataset using the robust multichip average (RMA) method [25], which is implemented in the BioConductor package ‘Affy’ [26]. The ‘Affy’ package was also used to generate RNA digestion plots which allow any 5′ to 3′ trend to be visualized. A linear regression of expression values on the logarithm (base 2) of slope of the RNA digestion plots for each probeset was performed and the residuals from the regression were assigned as expression values for further analysis. This transformation effectively corrected for any systematic error in the data introduced by 3′ signal bias and significantly improved the quality of the data (see Supplementary Information S1). Following transformation, all 54647 probes were included in the analysis. The data have been submitted to GEO (www.ncbi.nlm.nih.gov/geo/), accession numbers GSM318410-GSM318441; series record GSE12679.

#### Demographics

Demographic variables for the disease-based analysis are shown in Tables 1 & 2. No significant differences between patients and controls were found for pH, PMI or age in either cell type. The distribution of demographic variables for the cell-type analysis can be found in Supplementary Information S1.

**Table 1:** Endothelial sample demographics.

Group (n)	Control (7)	Schizophrenia (9)	*p* value
Age (yrs; mean±SD)	42.7±7.8	43.2±10.6	0.917
Gender (*n;* F/M)	1/6	3/6	n/a
pH (mean±SD)	6.6±0.2	6.5±0.3	0.363
PMI (hours; mean±SD)	20.3±10.4	29.6±10.1	0.094
Diagnosis (*n;* paranoid/undifferentiated)	n/a	2/7	n/a
Medicated at TOD (n; yes/no)	0	8/1	n/a
Lifetime medication (fluphenazine mg equiv; mean±SD)	0	112838±154662	n/a

Demographic variables for endothelial samples included in the disease-based analysis (F: female; M: male; PMI: post mortem interval; SD: standard deviation; TOD: time of death).

**Table 2:** Neuronal sample demographics.

Group (n)	Control (6)	Schizophrenia (5)	*p* value
Age (yrs; mean±SD)	41.2±7.2	45.0±5.4	0.353
Gender (*n;* F/M)	2/4	2/3	n/a
pH (mean±SD)	6.6±0.2	6.6±0.1	0.682
PMI (hours; mean±SD)	30.0±18.6	28.4±7.9	0.862
Diagnosis (*n;* paranoid/undifferentiated)	n/a	1/4	n/a
Medicated at TOD (*n;* yes/no)	0	4/1	n/a
Lifetime medication (fluphenazine mg equiv; mean±SD)	0	44010±35056	n/a

Demographic variables for neuronal samples included in the disease-based analysis (F: female; M: male; PMI: post mortem interval; SD: standard deviation; TOD: time of death).

#### LIMMA

A Bayesian moderated t-test was applied to identify differentially expressed genes as implemented in the LIMMA (linear models for microarray analysis) package [27] from Bioconductor. Firstly, pre-processed Affymetrix datasets were collapsed to the probeset with the maximum expression value for each gene using the Gene Set Enrichment Analysis software (details below). Differentially expressed genes were then identified using the LIMMA package and raw p-values were adjusted for multiple hypothesis testing using the false discovery rate (FDR) method of Benjamini and Hochberg [28].

#### Functional profiling

Two complimentary approaches to gene set analysis were employed. Both methods investigated all “biological process” categories as defined by the Gene Ontology consortium (GO). The default GSEA significance threshold of *q*<0.25 (after controlling the false discovery rate) was used for all functional analyses.

#### GSEA

The GSEA algorithm examines a ranked list of all genes on the chip, and identifies whether members of a gene category are enriched at either the top or bottom, using a modified Kolmogorov-Smirnov statistic. GSEA was carried out following the recommendations of the authors [29], [30]. Pre-processed expression data was inputted to the GSEA software and collapsed to the probe with the maximum expression level for each gene prior to analysis. Genes were ranked by fold change calculated using the “difference of class means” metric implemented in the GSEA software, such that genes ranked towards the top of the list are considered enriched in one sample group and genes ranked at the bottom are considered enriched in the other. Enrichment scores were calculated using the weighted enrichment statistic, and significance levels calculated by permuting phenotype labels 1000 times. Gene set size filters were set to exclude gene sets containing fewer than 25, or greater than 500 members. All other parameters were set to GSEA defaults.

In the schizophrenia versus control analysis the gene sets investigated comprised the complete list of human biological process categories present on the U133 Plus 2.0 array as defined by the GO consortium (subject to filters as described above; around 3000 categories in total). Some categories represent closely related functions and in addition, multifunctional genes may be annotated in more than one category. GSEA examines each gene set independently and hence multiple categories annotated to the same or similar genes can arise due to the hierarchical nature of the GO database. We therefore used the leading edge analysis tool within GSEA to identify related sets, i.e. those in which the significance is driven by an overlapping subset of genes (the “leading edge”).

#### OntoExpress

The OntoExpress software uses an algorithm which examines a predetermined list of significant genes and identifies categories of genes which are over or under represented in this list relative to their representation on the entire chip. Following LIMMA analysis genes were ranked in order of t statistic. A list of the top 2% of genes most significantly up- and down-regulated (this included 402 genes from the Affymetrix dataset corresponding to a *p* value cutoff of 0.027 among the upregulated genes and 0.047 among the downregulated genes) were analysed separately using OntoExpress, using default settings (hypergeometric distribution and FDR (Benjamini-Hochberg) correction) and the Affymetrix human HG-U133 Plus 2.0 array as reference. Categories which had at least 3 members were considered in the analysis.

### Codelink arrays

#### Data preprocessing

Image analysis and feature extraction was performed using the proprietary Amersham CodeLink Bioarray software (GE Healthcare). A flag-based noise filter was applied such that probes were retained for further analysis which had a “good” flag in a minimum number of arrays corresponding to the smallest sample group tested (eg where there were 12 control and 12 schizophrenia samples, the filter was set to retain probes which had a “good” flag in at least 12 samples). This step was carried out independently for the cell-type and disease analyses. The spot mean signal intensities for probes passing the filter were quantile normalized [31] to generate gene expression measures. Outlier removal was carried out based on a correlation matrix generated from all possible pairwise comparisons between arrays using Pearson's product-moment correlation coefficient as the metric. Poorly correlating arrays were removed from the analysis. After removing outliers, the flag-based noise filter and normalisation process were re-applied. The final dataset for the cell type analysis (all samples) contained 10487 probes. For the disease-based analysis endothelial and neuronal samples were normalised separately and the final dataset contained 8846 probes for the endothelial samples and 14262 probes for the neuronal samples. Due to the much reduced number of probes on the Codelink arrays compared to the Affymetrix arrays, Codelink arrays were used solely for validation purposes and not for the primary analysis.

### Cross platform validation

Data were cross-validated between array platforms using GSEA and the method of Cheadle *et al* (2006) [32]. This method was developed to examine the entire dataset, taking into account the differences in absolute mRNA quantitation which often occur between array platforms. Following one microarray analysis, the most significantly altered genes are used to create a category. Data from an alternative microarray platform are then probed with this category. If the data are reproducible in the second platform, the category should be significantly enriched in the predicted direction. Datasets were collapsed to the maximum probe level per gene using GSEA, filtered to those genes which were present across both platforms and ranked using the GSEA metric “difference of class means”. The 200 top and bottom ranking genes from each array platform were each used to create a gene set. We then determined whether the top ranking genes from each platform showed enrichment in the same direction in the other platform, using the GSEA parameters described above.

## Results

### Cell type analysis

**Figure 3:**
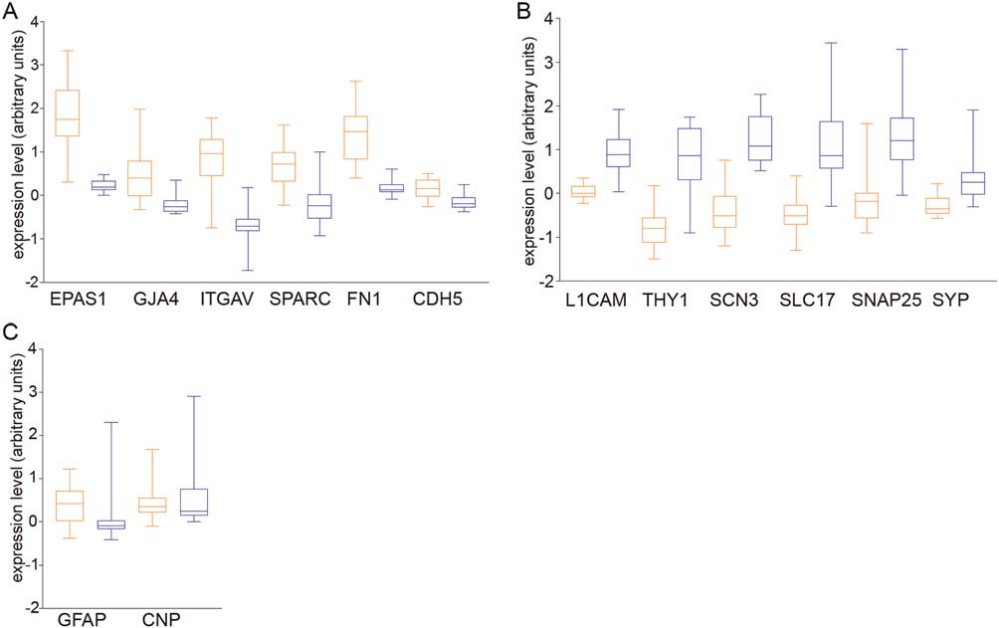
Characteristic endothelial and neuronal genes. Boxplots showing the expression levels (arbitrary units, derived from Affymetrix Genechips) in endothelial (red) and neuronal (blue) samples of a panel of genes known to be preferentially expressed in either (a) endothelial cells or (b) neurons in brain tissue, plus (c) two markers known to be expressed in other cell types.

As an initial assessment of the biological validity of the data, we compiled a list of biological processes, as defined by the Gene Ontology Consortium, likely to be specific to one cell type or the other, using the search terms “neuron”, ”neurotransmitter” and “synapse” plus “endothelial”, and “angiogenesis”. Due to the small number of categories found to be endothelial-related, we also included cell proliferation as an endothelial-related category, as there is no published evidence for neurogenesis in the adult prefrontal cortex [33]. These categories were tested for enrichment in the Affymetrix data using GSEA. All endothelial categories were found to be significantly enriched in the endothelial data, and all neuronal categories were found to be significantly enriched in the neuronal data (*q*<0.25; Table 3). Additionally we chose a panel of six genes expected from the literature to be preferentially expressed in one cell type or the other in brain tissue, and investigated whether these were found differentially expressed between cell types using our methods. In the Affymetrix dataset, all six genes considered to differentiate endothelial cells from neurons (fibronectin (FN1), osteonectin (SPARC), integrin alpha5 (ITGAV), vascular endothelial cadherin (CDH5), endothelial PAS domain protein 1 (EPAS1), gap junction protein alpha4 (GJA4)) were significantly upregulated in our endothelial cell samples, and all six of those chosen to be neuronal differentiators (neural cell adhesion molecule L1 (L1CAM), synaptosomal protein 25 (SNAP25), synaptophysin (SYP), voltage gated sodium channel type IIIbeta (SCN3B), vesicular glutamate transporter (SLC17A7), Thy-1 cell surface antigen (THY1)) were significantly upregulated in our neuronal samples (Fig 3, Table 4). Furthermore, we examined the expression of markers of other cell types: GFAP, an astrocyte marker, and CNP, a marker of oligodendrocytes. These genes showed low expression values, and did not show differential expression between cell types. The method of Cheadle et al [32] was used to cross validate the entire dataset between chip platforms. In all cases both platforms reflected similar changes for genes with the greatest differential expression between endothelial cells and neurons (Table 5). Furthermore, four of each of the neuronal and endothelial markers were also detected in the Codelink dataset; only one failed to cross validate between datasets (Table 4).

**Table 3:** Results of cell type comparison–gene categories.

GO ID	Category Name	Cell type	NES	FDR *q* value
GO:0001525	angiogenesis	E	1.49	0.028
GO:0008283	cell proliferation	E	1.51	0.037
GO:0045765	regulation of angiogenesis	E	1.40	0.046
GO:0048666	neuron development	N	−1.42	0.055
GO:0030182	neuron differentiation	N	−1.43	0.060
GO:0019226	transmission of nerve impulse	N	−1.45	0.061
GO:0048699	generation of neurons	N	−1.45	0.075
GO:0048667	neuron morphogenesis during differentiation	N	−1.46	0.092
GO:0007269	neurotransmitter secretion	N	−1.56	0.093
GO:0007268	synaptic transmission	N	−1.48	0.118
GO:0042551	neuron maturation	N	−1.23	0.166
GO:0001505	regulation of neurotransmitter levels	N	−1.26	0.169
GO:0050808	synapse organization and biogenesis	N	−1.24	0.173
GO:0006836	neurotransmitter transport	N	−1.13	0.241

GSEA results for categories characteristic of either endothelial cells (E) or neurons (N). Positive normalized enrichment scores (NES) indicate categories enriched in endothelial samples, negative NES indicate categories enriched in neuronal categories. All endothelial categories showed significant enrichment in the endothelial samples and all neuronal categories showed significant enrichment in the neuronal samples (*q*<0.25).

**Table 4:** Results of cell type comparison–individual genes.

Gene symbol	Gene name	Cell type	Affymetrix		Codelink	
			Fold change	*q* value	Fold change	*q* value
EPAS1	Endothelial PAS domain protein 1	E	3.09	<0.0001	0.93	0.5568
GJA4	Gap junction protein alpha4	E	1.62	0.0116	1.18	0.0645
ITGAV	Integrin alpha5	E	2.95	<0.0001	*Not detected*	
SPARC	Osteonectin	E	1.87	0.0012	1.56	0.0003
CDH5	Vascular endothelial cadherin	E	1.24	0.0124	*Not detected*	
FN1	Fibronectin	E	2.36	<0.0001	1.99	<0.0001
L1CAM	Neural cell adhesion molecule L1	N	−1.84	0.0001	*Not detected*	
THY1	Thy-1 cell surface antigen	N	−2.94	<0.0001	−1.99	0.0045
SCN3B	Voltage gated sodium channel type IIIbeta	N	−3.04	<0.0001	*Not detected*	
SLC17A7	Vesicular glutamate transporter	N	−3.14	<0.0001	−1.32	0.0011
SNAP25	Synaptosomal protein 25	N	−2.75	0.0007	−1.32	0.0423
SYP	Synaptophysin	N	−1.54	0.0121	−1.20	0.0009
GFAP	Glial fibrillary acidic protein	A	1.22	0.4727	*Not detected*	
CNP	2′,3′-cyclic nucleotide 3′ phosphodiesterase	O	−1.07	0.8321	*Not detected*	

Differential expression of genes previously shown to be preferentially expressed in neurons (N), endothelial cells (E), astrocytes (A), or oligodendrocytes. Fold changes are calculated as endothelial cells relative to neurons. Of the genes which were present across both array platforms, only EPAS1 was not significant in both.

**Table 5:** Results of cross platform validation of the cell type comparison.

CODELINK DATA		
*Categories tested*	*Enrichment score*	*FDR q-value*
*AFFY_ENDO*	1.63	0.001
*AFFY_NEURON*	−1.32	0.148

AFFYMETRIX DATA		
*Categories tested*	*Enrichment score*	*FDR q-value*
*CL_ENDO*	1.76	0.000
*CL_NEURON*	−1.3	0.171

Based on the Affymetrix data, the 200 genes whose expression was the highest in endothelial cells compared to neurons (AFFY_ENDO), and the 200 genes whose expression was the highest in neurons compared to endothelial cells (AFFY_NEURON) were used to create categories. These Affymetrix-derived categories were then investigated in the ranked gene list (endothelial cells vs neurons) generated using the Codelink platform. A positive enrichment score indicates enrichment in endothelial cells, and a negative score indicates enrichment in neurons. The categories were all found to be significantly enriched in the predicted direction in the Codelink data (*q*<0.25). The converse procedure was carried out testing categories generated from the Codelink data (CL_ENDO, CL_NEURON) on the Affymetrix platform, with comparable results.

**Figure 4:**
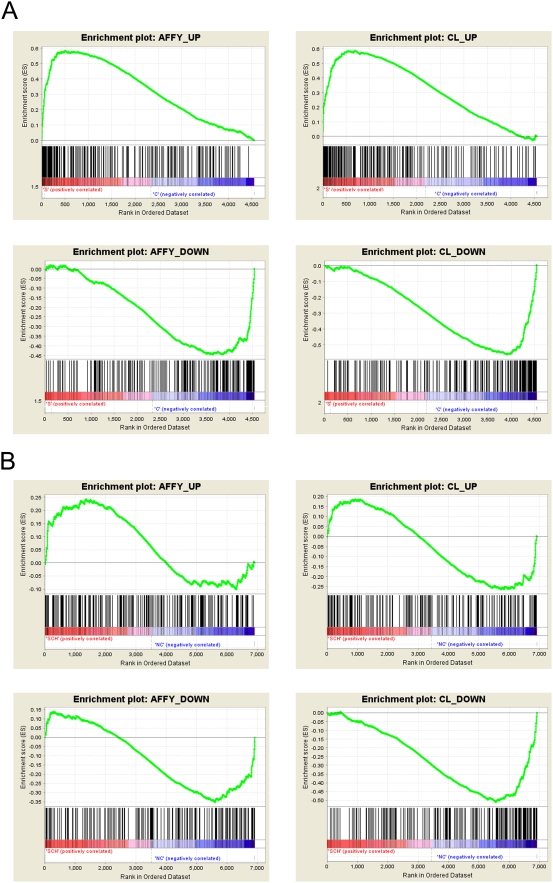
Results of cross-platform validation of the schizophrenia vs control analysis for (a) endothelial cells and (b) neurons. The figures were created by the GSEA software and plot the running enrichment score (ES) which reflects the degree to which a gene set is overrepresented at the top or bottom of a ranked list of genes. The score at the peak of the plot (the score furthest from 0.0) is the ES for the gene set. The position of individual members of the gene set in the ranked list is indicated by vertical lines.

Having established that biologically valid differences between cell types could be detected in the dataset, we moved on to investigate whether differences could be detected between schizophrenia patients and controls in either cell type. LIMMA analysis revealed 1156 genes significantly differentially expressed in endothelial cells and 803 in neurons at *p*<0.05; however, when correction for multiple hypothesis testing was applied, no genes reached significance. This is likely to be explained by the small sample number and relative subtlety of the expected disease-related changes. In order to determine whether the disease signal could be validated using an alternative methodology, we applied the method of Cheadle et al [32] to validate across chip platforms. In the endothelial dataset, a set of genes whose expression was the most different between schizophrenia and control on one chip platform were found to be significantly enriched in the predicted direction on the other platform (Table 6, Fig 4), suggesting that a reproducible disease signal could be detected in the endothelial samples. However, in the neuronal dataset, alterations in gene expression between schizophrenia and control could not be validated across chip platforms (Table 7). This indicates that using this methodology, no technically robust disease-related alterations could be detected in neurons from the schizophrenia samples, in contrast to the endothelial cells.

**Table 6:** Results of cross platform validation of the schizophrenia vs control comparison for endothelial cells.

CODELINK DATA		
*Categories tested*	*Enrichment score*	*FDR q-value*
*AFFY_UP (SZ)*	1.5	0.013
*AFFY_DOWN (C)*	−1.44	0.044

AFFYMETRIX DATA		
*Categories tested*	*Enrichment score*	*FDR q-value*
*CL_UP (SZ)*	1.65	0.000
*CL_DOWN (C)*	−1.69	0.000

Based on the Affymetrix data, the 200 genes whose expression was most upregulated in schizophrenia (AFFY_UP), and the 200 genes whose expression was the most downregulated in schizophrenia (AFFY_DOWN) were used to create categories for analysis in the ranked list (schizophrenia vs control) derived from the Codelink platform. A positive enrichment score indicates enrichment in schizophrenia samples, and a negative enrichment score indicates enrichment in control samples. These were all found to be significantly enriched in the expected direction in the Codelink data (*q*<0.25). The converse procedure was carried out testing categories generated from the Codelink data (CL_UP, CL_DOWN) on the Affymetrix platform, with comparable results.

**Table 7:** Results of cross platform validation of the schizophrenia vs control comparison for neurons.

CODELINK DATA		
*Categories tested*	*Enrichment score*	*FDR q-value*
*AFFY_UP (SZ)*	0.77	0.734
*AFFY_DOWN (C)*	−1.15	0.283

AFFYMETRIX DATA		
*Categories tested*	*Enrichment score*	*FDR q-value*
*CL_UP (SZ)*	−1.36	0.132
*CL_DOWN (C)*	−1.69	0.589

The same procedure as described in Table 6 (endothelial cells) was carried out on data from the neuronal samples. In only one of the four tests was the category derived from one platform significantly enriched in the expected direction on the other platform.

In order to further characterise the disease signal in endothelial cells, we investigated alterations in functional categories of genes, a statistically more powerful approach than considering individual gene changes. Numerous approaches to functional profiling of gene expression data exist, which rely on different computational approaches and assumptions. In the present study, we employed two algorithms. The first, GSEA, looks for enrichment of genes in a category at the top or bottom of a ranked list based on a modified Kolmogorov-Smirnov statistic, and has been developed specifically for the detection of biological differences which may be modest relative to technical noise. The second, OntoExpress, identifies categories of genes which are over or under represented in a list of significant genes relative to their representation on the entire chip. Some differences are expected due to the differences between the algorithms [34], but a truly robust finding should be detectable using either method.

62 categories were downregulated in schizophrenia endothelial samples using GSEA and 33 using OntoExpress. The majority of results were comparable regardless of the algorithm used (Table 8), with a small number of exceptions: endothelial-specific and developmental categories were only found significant with GSEA and not OntoExpress, and categories relating to transcriptional and translational processes were identified with OntoExpress but not GSEA. No categories were significantly upregulated, which reflects the bias towards downregulated genes seen in the data (not shown).

**Table 8:** Results of functional profiling of the endothelial cell data.

GSEA				OntoExpress		
GO ID	Name	Size	FDR q value	GO ID	Name	FDR q value
**RESPONSE TO STIMULUS**						
GO0007606	sensory perception of chemical stimulus	105	0.060	GO:0007600	sensory perception	0.097
GO0007608	sensory perception of smell	86	0.114			
GO0009581	detection of external stimulus	32	0.147			
**IMMUNE SYSTEM**						
GO0051240	positive regulation of organismal physiological process	52	0.121	GO:0006952	defense response	0.028
GO0050867	positive regulation of cell activation	25	0.132	GO:0006955	immune response	0.038
GO0046651	lymphocyte proliferation	25	0.145	GO:0030154	cell differentiation	0.121
GO0050778	positive regulation of immune response	48	0.148			
GO0046649	lymphocyte activation	82	0.149			
GO0042113	B cell activation	32	0.150			
GO0016066	cellular defense response (sensu Vertebrata)	27	0.156			
GO0050776	regulation of immune response	73	0.161			
GO0050865	regulation of cell activation	40	0.164			
GO0045321	immune cell activation	96	0.193			
GO0030098	lymphocyte differentiation	34	0.199			
GO0051249	regulation of lymphocyte activation	38	0.201			
GO0001775	cell activation	97	0.202			
GO0006959	humoral immune response	137	0.140			
GO0016064	humoral defense mechanism (sensu Vertebrata)	100	0.212			
GO:0019735	antimicrobial humoral response (sensu Vertebrata)	75	0.228			
GO0019730	cytokine production	78	0.249			
GO0009613	response to pest, pathogen or parasite	479	0.124	GO:0006954	inflammatory response	0.089
GO0009611	response to wounding	362	0.129			
GO0006954	inflammatory response	198	0.132			
GO0009605	response to external stimulus	460	0.146			
GO0019882	antigen presentation	39	0.139			
GO0030333	antigen processing	32	0.150			
GO0045087	innate immune response	55	0.106			
GO0042742	defense response to bacteria	53	0.117			
GO0009615	response to virus	66	0.156	GO:0009615	response to virus	0.034
GO0006968	cellular defense response	93	0.174	GO:0006968	cellular defense response	0.065
**FERTILIZATION**						
GO0007338	fertilization (sensu Metazoa)	35	0.158	GO:0007283	spermatogenesis	0.037
GO0009566	fertilization	36	0.161			
**MOTILITY**						
GO0051270	regulation of cell motility	28	0.144	GO:0006928	cell motility	0.097
GO0040012	regulation of locomotion	28	0.149	GO:0030155	regulation of cell adhesion	0.007
GO0030334	regulation of cell migration	28	0.153			
GO:0016477	cell migration	95	0.226			
GO0007610	behavior	175	0.137	GO:0010033	response to organic substance	0.001
GO0042330	taxis	107	0.144			
GO0007626	locomotory behavior	112	0.149			
GO0006935	chemotaxis	107	0.155			
GO:0042221	response to chemical stimulus	310	0.223			
**ION TRANSPORT**						
GO0030005	di-, tri-valent inorganic cation homeostasis	89	0.154	GO:0006811	ion transport	0.093
GO0006875	metal ion homeostasis	96	0.157	GO:0006810	transport	0.168
GO0030003	cation homeostasis	102	0.199			
GO0006817	phosphate transport	76	0.196			
**NEGATIVE REGULATION OF CELL PROLIFERATION**						
GO0008285	negative regulation of cell proliferation	145	0.147	GO:0016049	cell growth	0.036
				GO:0006915	apoptosis	0.066
**DNA METABOLISM**						
GO0051052	regulation of DNA metabolism	31	0.161	GO:0006259	DNA metabolism	0.003
				GO:0006281	DNA repair	0.043
**PROTEIN MODIFICATION**						
GO0045860	positive regulation of protein kinase activity	53	0.146	GO:0006464	protein modification	0.079
GO0045859	regulation of protein kinase activity	123	0.161			
GO0043549	regulation of kinase activity	123	0.165			
GO0051338	regulation of transferase activity	123	0.157			
GO0000079	regulation of cyclin-dependent protein kinase activity	38	0.167			
GO0051347	positive regulation of transferase activity	53	0.150			
**ENDOTHELIAL-SPECIFIC**						
GO0001525	angiogenesis	61	0.246			
GO0008217	blood pressure regulation	29	0.175			
**DEVELOPMENT**						
GO0007398	ectoderm development	85	0.119			
GO0008544	epidermis development	77	0.144			
GO0009888	tissue development	171	0.177			
GO0007519	striated muscle development	38	0.148			
GO0007517	muscle development	98	0.198			
**OTHER**						
GO0000270	peptidoglycan metabolism	26	0.129			
**PROTEIN ASSEMBLY/TRANSPORT**						
				GO:0006461	protein complex assembly	0.094
				GO:0006508	proteolysis and peptidolysis	0.100
				GO:0015031	protein transport	0.077
				GO:0006886	intracellular protein transport	0.196
**SIGNAL TRANSDUCTION**						
				GO:0007186	G-protein coupled receptor protein signaling pathway	0.079
				GO:0007165	signal transduction	0.006
**TRANSCRIPTION/TRANSLATION**						
				GO:0045449	regulation of transcription	0.032
				GO:0006445	regulation of translation	0.043
				GO:0008380	RNA splicing	0.063
				GO:0006396	RNA processing	0.080
				GO:0006260	DNA replication	0.195
				GO:0006355	regulation of transcription, DNA-dependent	0.197
				GO:0006350	transcription	0.185

Gene function categories downregulated in endothelial cells from schizophrenia patients using GSEA (left side) and OntoExpress (right side). Related gene sets as defined by GSEA leading edge analysis are grouped together in column 2, and categories are further grouped by theme. Equivalent categories (identical or closely related in the Gene Ontology) identified by OntoExpress, are indicated in columns 5 and 6.

## Discussion

In the present study we show molecular alterations in the vasculature of schizophrenia patients. Based on these data alone it is not possible to infer whether alterations in RNA expression reflect a functional impairment in the blood brain barrier. Nonetheless, the results of gene expression profiling indicate a downregulation of genes involved in ion transport, cell proliferation and adhesion, which are consistent with such an impairment. Furthermore, downregulation of genes related to immune system function was identified, including GO:0006954 “inflammatory response” which was identified using both OntoExpress and GSEA. Hanson and Gottesman have proposed inflammation of the cerebral microvasculature as the source of blood-brain barrier dysfunction in schizophrenia, with systemic effects [9]. However the data from this study, and other studies from our laboratory on T cell function in schizophrenia [35], point more towards a hypo-inflammatory state in schizophrenic patients. This is consistent with a growing body of evidence in the field, including the negative association between schizophrenia and rheumatoid arthritis [36]–[38], lower antibody reactions to vaccination [39], and decreased skin sensitivity to the type IV antigen test [40]. However, schizophrenia has been positively linked to other auto-immune disorders [41]. At the molecular level, increased levels of acute phase proteins have been reported in schizophrenia pointing towards a pro-inflammatory state [42], [43], and conflicting data exists on the role of inflammatory cytokines in the disorder [44]. A potential explanation for these apparently opposing results is overall dysregulation of inflammation, leading to an inappropriate response (either too much or too little inflammation) depending on the stimulus and site. Such a dysfunction in the blood brain barrier is consistent with the broader implications of Hanson & Gottesman's hypothesis, and could result in slower response and lower resistance to brain injury/insult, affecting the regulation of supply of substances to the brain. Furthermore, abnormal inflammatory processes may have downstream effects on angiogenesis [45], and thus may further impact vascular abnormalities. However, conclusions cannot be firmly drawn without further in vivo study of the microvasculature in schizophrenia patients.

The blood brain barrier is composed of endothelial cells, pericytes, astrocyte end-feet and neuronal processes. A method for isolating pure vascular endothelium has been developed [46]; however, as this method is specific for endothelial cells and involves numerous steps, its use would preclude comparison of the resulting cell population with neurons and other cell types. Furthermore, cerebral microvascular function arises from the interactions between its various components, therefore global data for the intact microvessel is of greater interest. In this context, our result of downregulation of genes in the GO category “spermatogenesis” is of interest as numerous functional links exist between the blood-brain barrier and the blood-testis barrier, including shared properties of astrocytes and oligodendrocytes and the Sertoli and Leydig cells of the testis [47], [48]. We speculate that this result may indicate altered gene expression in non-endothelial components of the blood-brain barrier. Interestingly, a recent study has found a decreased number of oligodendrocytes per unit length of capillary in post mortem prefrontal cortex from schizophrenia patients [49] which provides a potential explanation for some of the results seen here. Further investigation of these cell types is clearly warranted.

Laser capture microdissection is a technically challenging method due to the small amounts of tissue and the variability that may be introduced at various stages of the analytical process, compounded by the effects of working with post mortem human tissue, and the present work involved extensive optimization and validation of the methods prior to study commencement. In the present study, although cell-type specific changes could be detected in neurons, no schizophrenia-related alterations could be reliably detected in neurons collected from the same brain region as the endothelial cells (dorsolateral prefrontal cortex). Although we (data not shown) and others [50]–[52] have found that RNA amplification gives reproducible results, it does result in loss and/or truncation of transcripts, leading to a smaller number of high-quality arrays than would otherwise be expected, and furthermore the final mRNA population analysed will not contain the full range of information found in the original sample [50], [53], [54]. Thus in this context the neuronal result cannot be considered a true negative. Although no directly comparable study has been carried out, there is much evidence for neuronal alterations in schizophrenia at the molecular and structural level [55], including data derived using similar methods in the same [56], [57] and other [2] brain regions. However, as the endothelial data were collected in an identical manner, the results do provide compelling preliminary evidence for molecular alterations in the microvasculature of schizophrenia patients. More targeted and functional studies of the blood-brain barrier, including investigation of its subcomponents, are now required, and further investigation is necessary to determine whether, if proven, blood-brain barrier dysfunction can directly explain the impairment in glucose utilization in the brains of schizophrenic patients. The potential effects of antipsychotic medication on the blood brain barrier should also be assessed. Furthermore, investigation of other cell types such as astrocytes, and further investigation of peripheral tissues, is key to elucidating the role of metabolic abnormalities in the pathophysiology of schizophrenia.

## Supporting Information

Supplementary Information S1 A document containing demographics variables for the cell type analysis, and additional details of the microarray data processing
(0.05 MB DOC)

Figure S1 RNA 5′ -3′ signal bias. (a) RNA digestion plot showing signal from probes decreases with distance of target sequence from 3′ end of transcript. Note the inter-chip variability in the gradient of the curves. (b) The degree of RNA 5′-3′ signal bias within a chip, as measured by the slope of RNA digestion curve, displays strong positive correlation with the number of probe-sets on the chip which are flagged as present.
(7.00 MB TIF)

Figure S2 Detection and removal of RNA 5 -3signal bias from Affymetrix GeneChip data on the expression profiles of endothelial cells (E) and neurons (N). (a) PCA of RMA expression data reveals that the major component of the variation in the data (PC1) is not related to differential expression between endothelial cells and neurons. (b) PC1 of the RMA expression data shows strong correlation with the RNA 5 -3signal bias within each chip. The slope of a chip's RNA digestion curve was used as the measure of 5 -3signal bias. (c) Following a transformation to remove 5 -3signal bias (see text for details), the major source of variation in the data set is now differential gene expression between the two cell types, which are now clearly separable on PC1. (d) PC1 of transformed data is not correlated with the 5 -3signal bias within a chip.
(8.23 MB TIF)

Figure S3 Number of probesets on the Affymetrix GeneChip detecting differential ex- pression between endothelial cells and neurons at a range of false discovery rates (FDR), before and after a correction was applied for 5′-3′ signal bias. For details of systematic bias and correction, see text.
(6.77 MB TIF)
