# Supplementary material for: The Cerebral Microvasculature in Schizophrenia: A Laser Capture Microdissection Study
Source: PLoS One. 2008 Dec 17;3(12):e3964. doi: 10.1371/journal.pone.0003964 (PMC2597747; doi:10.1371/journal.pone.0003964)
Supplement: Supplementary Information S1 — A document containing demographics variables for the cell type analysis, and additional details of the microarray data processing (0.05 MB DOC) [file pone.0003964.s001.doc]

**Supplementary information for LCM paper**

Supplementary Table 1: Demographics of samples included in the cell type analysis after outlier removal on both array platforms (F: female; M: male; PMI: post mortem interval; SD: standard deviation).

| **Demographic (n)** | **Endothelial (16)** | **Neuronal (16)** |
| --- | --- | --- |
| Diagnosis (*n;* NC/SZ/BPD) | 7/9/0 | 6/5/5 |
| Age (years; mean ±SD) | 43.0±9.16 | 43.1±6.19 |
| Gender (*n;* F/M) | 4/12 | 5/11 |
| pH (mean ±SD) | 6.5±0.26 | 6.60±0.14 |
| PMI (hours; mean ±SD) | 25.5±10.98 | 28.9±11.82 |

**Optimisation of data analysis framework**

The following section describes the technical optimisation steps that were used to develop the preprocessing methods for data derived from laser captured samples analysed using Affymetrix Genechips. This optimisation work was carried out on a larger sample set than that used in the main manuscript, which included subjects with bipolar disorder

*Affymetrix data preprocessing*

The process of RNA amplification usually results in truncation of the 5’ end of the transcript {McClintick, 2003 #73}. Since Affymetrix probe-sets comprise multiple probes matching sequence at various positions along the target transcript, it was possible to use the Affymetrix data to investigate this phenomenon of 3 signal bias. The ’Affy’ package {Irizarry, 2005 #27} from the BioConductor project provides RNA digestion plots for the assessment of RNA 5 -3signal bias. In the RNA digestion plots, individual probes in a probe-set are ordered by location relative to the 5 end of the targeted RNA molecule. For each chip, probe intensities are averaged by their location within the probe-set, with the average taken

over all probe-sets. Mean probe intensities are then standardised and plotted against the probe’s position within the probe-set, allowing any 5 to 3 trend to be visualised **(figure S1a)**.

Next, a ‘present’, ‘marginal’, or ‘absent’ flag was assigned to each probe set on each GeneChip using the ‘mas5calls’ function from the BioConductor package ‘Affy’ {Irizarry, 2005 #27}. The flags provide a measure of confidence in the signal obtained from the probesets. A ‘present’ flag indicates that a probeset has reliably detected its target transcript(s), whereas an ‘absent’ or ‘marginal’ flag suggests that the signal from the probe set may merely be noise. The proportion of probe sets on each GeneChip called ‘present’ ranged from 5 - 27% (mean =13%). This metric is typically used as an index of hybridisation success, with high quality assays yielding a higher proportion of ‘present’ calls than low

quality assays. Therefore, it was surprising to find that the logarithm of the number of probesets flagged ‘present’ on a chip showed a strong positive correlation (r^2^ = 0.93)

with a measure of 3 signal bias; i.e. the slope of the chip’s RNA digestion plot **(figure S1b)**. The cause of this apparently paradoxical relationship is far from clear. However, it has been noted in a previous study{Ryan, 2004 #22} that RNA digestion plots for good quality hybridisations do display 3’ signal bias whereas plots for some poor quality hybridisations do not.

Any systematic bias remaining after normalization can be assessed using multivariate statistics. Principal components analysis (PCA) is a relatively simple method for identifying the major trends of variation in a data set and is implemented in the R function ‘prcomp’. PCA takes the original variables (in this case expression measures for 54 675 probe sets) and transforms them to a small number of uncorrelated variables termed principal components (the number of principal components equals the number of samples). An assumption of PCA is that many of the original variables are correlated and thus by eliminating this redundancy, the variation in the data can be described by a small number of principal components. Principal components are extracted from the data sequentially, with principal component 1 (PC1) representing the largest amount of variation in the data, followed by principal component 2 (PC2) representing the next largest amount of variation, and so on. Figure S2a is a scatterplot of the first two principal components for the Affymetrix expression matrix of 36 chips and 54 675 probe sets. PC1 accounts for 24% of the variance in the data set and PC2 for 10%. If we assume that the main biological signal in the microarray data is the difference between the cell types,

then in the absence of systematic measurement error, we would expect the two cell types to separate along PC1, the component describing most variance.

However, this is not the case. For PC1, the samples actually showed more variance within cell type than between cell type. Since we could not attribute PC1 to a biological signal, we postulated a technical source of variation. Initially we investigated whether this systematic error could be eliminated if a different method was used to compute expression measures. We computed a further four expression measures, namely: microarray suite 5 (MAS5){Affymetrix, 2002 #77}, probe logarithmic intensity error estimation (PLIER){Affymetrix, 2005 #78}, GCRMA{Wu, 2005 #80} and variance stabilizing normalization (VSN){Huber, 2002 #81}, but none of these succeeded in reducing the apparent measurement error.

Further analysis of the RMA expression data revealed a strong negative correlation

(r2 = 0.879, p = 3.85×10^−16^) between PC1 and the logarithm of the number of ‘present’ flags on each chip. Moreover, an even stronger negative correlation (r2 = 0.920, p = 3.08× 10^−20^) was observed between PC1 and the slopes of the RNA digestion plots **(figure S2b)**. Thus much of the systematic bias in the microarray data could be described by both the number of ‘present’flags on each chip and the slopes of the RNA digestion plots. Of the two, the gradient of the RNA digestion plots was considered to be the better descriptor of systematic bias, because it was more strongly correlated with PC1.

This technical source of variation was corrected as follows: for each probe set we performed a linear regression of expression level on the gradients of the RNA digestion plots; the residuals from this linear regression were assigned as the expression values for the probe set. After this transformation the PCA was repeated and we found that the cell types now clearly separated on PC1, i.e. the major source of variance in the corrected data set was now attributable to biological signal **(figure S2c)**. What is more, in the corrected data set, PC1 is not correlated with the slopes of the RNA digestion plots

**(figure S2d)**. The underlying cause of the variation in 3 signal bias and its relationship

to the measured global gene expression profile is currently unknown, but is presumably some artifact of the experimental procedures. Whilst the regression method described above can help to remove this systematic error, it would be preferable to identify its origin and control it at source. Before proceeding with statistical tests for differential expression, the data from the pairs of technical replicate chips were summarised to single samples by taking the mean expression value for each probe-set. **Figure S3** demonstrates

the significant increase in assay sensitivity gained when measurement error is controlled using above linear transformation on the data. Genes differentially expressed between endothelial cells and neurons were identified using the Bayesian moderated t-test implemented in the BioConductor package LIMMA {Smyth, 2005 #30}. The number of false positives arising from multiple tests of hypothesis was controlled using the false discovery rate (FDR) procedure of Benjamini and Hochberg{Benjamini, 1995 #54}. At the conventional FDR threshold of α=0.05, reduction of the systematic bias resulted in a two-fold increase in the number of probe sets reporting differential expression between endothelial cells and neurons. This technique thus revealed more of the biological signal in the data.

**Figure Legends**

Fig. S1. RNA 5’ -3’ signal bias. (a) RNA digestion plot showing signal from probes

decreases with distance of target sequence from 3’ end of transcript. Note the inter-chip variability in the gradient of the curves. (b) The degree of RNA 5’-3’ signal

bias within a chip, as measured by the slope of RNA digestion curve, displays strong

positive correlation with the number of probe-sets on the chip which are flagged as

present.

**Fig S2.** Detection and removal of RNA 5 -3signal bias from Affymetrix

GeneChip data on the expression profiles of endothelial cells (E) and neurons (N).

(a) PCA of RMA expression data reveals that the major component of the variation

in the data (PC1) is not related to differential expression between endothelial cells

and neurons. (b) PC1 of the RMA expression data shows strong correlation with

the RNA 5 -3signal bias within each chip. The slope of a chip’s RNA digestion

curve was used as the measure of 5 -3signal bias. (c) Following a transformation to

remove 5 -3signal bias (see text for details), the major source of variation in the

data set is now differential gene expression between the two cell types, which are

now clearly separable on PC1. (d) PC1 of transformed data is not correlated with

the 5 -3signal bias within a chip.

**Fig S3** Number of probesets on the Affymetrix GeneChip detecting differential ex-

pression between endothelial cells and neurons at a range of false discovery rates

(FDR), before and after a correction was applied for 5’-3’ signal bias. For details of

systematic bias and correction, see text.
