# Supplementary figures and images for: The Cerebral Microvasculature in Schizophrenia: A Laser Capture Microdissection Study
Source: PLoS One. 2008 Dec 17;3(12):e3964. doi: 10.1371/journal.pone.0003964 (PMC2597747; doi:10.1371/journal.pone.0003964)

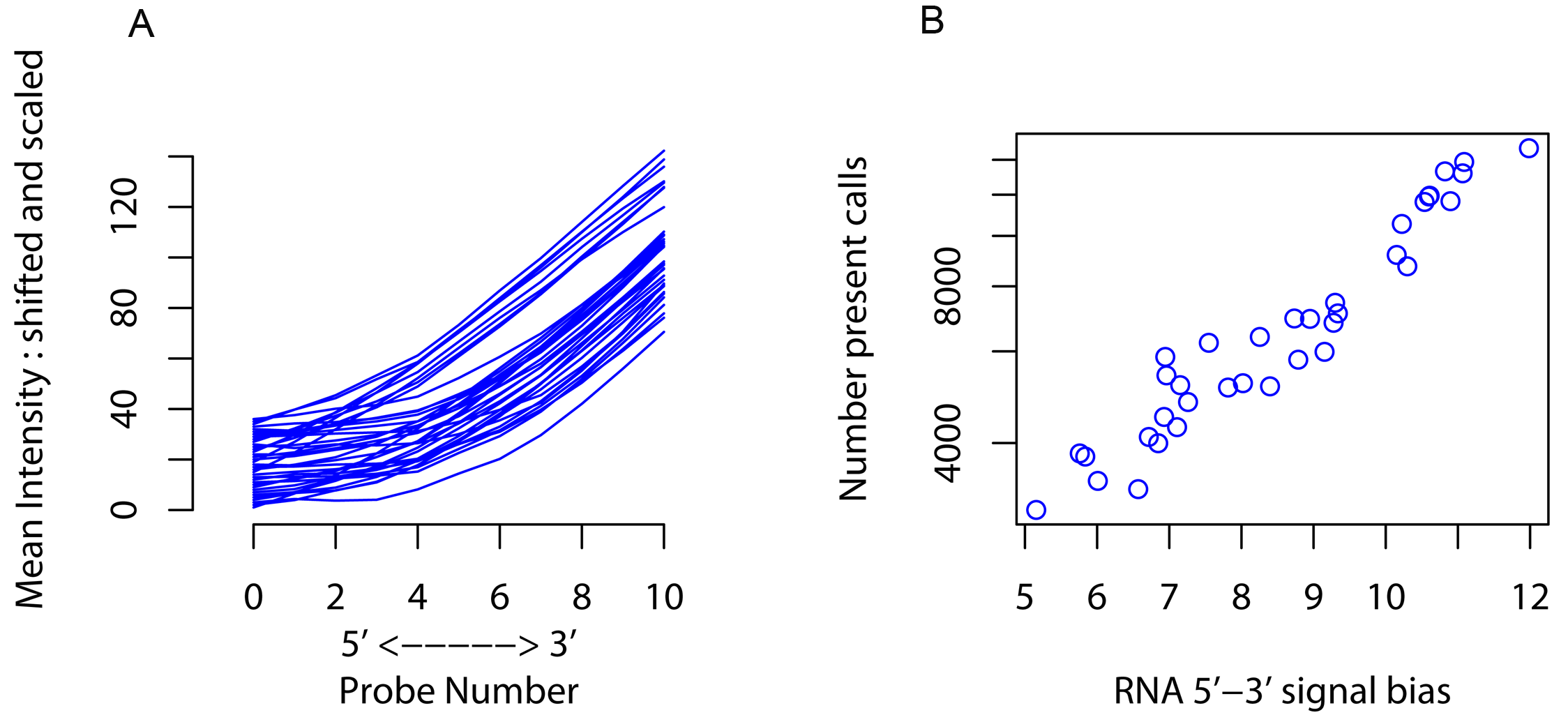

Supplement: Figure S1 — RNA 5′ -3′ signal bias. (a) RNA digestion plot showing signal from probes decreases with distance of target sequence from 3′ end of transcript. Note the inter-chip variability in the gradient of the curves. (b) The degree of RNA 5′-3′ signal bias within a chip, as measured by the slope of RNA digestion curve, displays strong positive correlation with the number of probe-sets on the chip which are flagged as present. (7.00 MB TIF) [file pone.0003964.s002.tif]

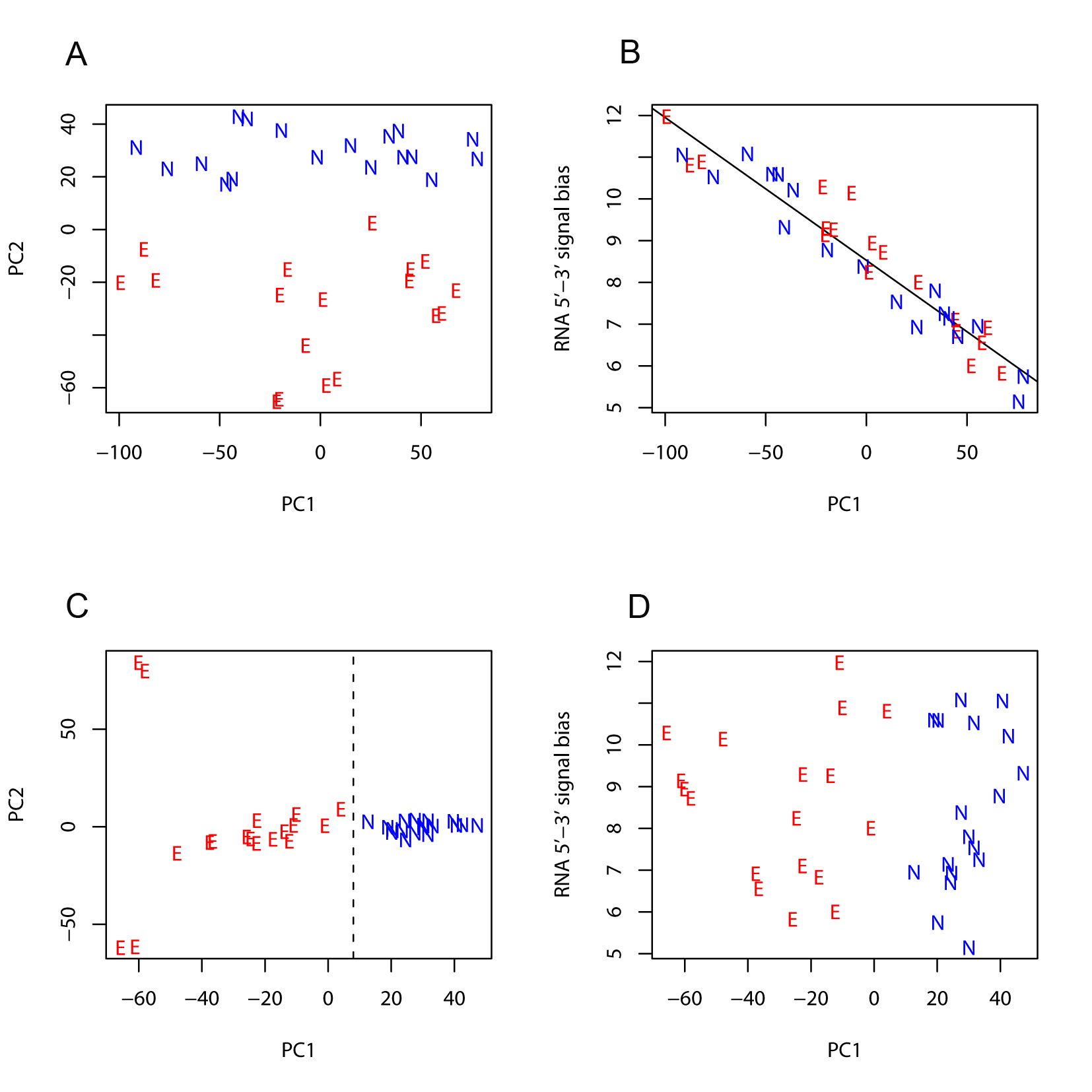

Supplement: Figure S2 — Detection and removal of RNA 5 -3signal bias from Affymetrix GeneChip data on the expression profiles of endothelial cells (E) and neurons (N). (a) PCA of RMA expression data reveals that the major component of the variation in the data (PC1) is not related to differential expression between endothelial cells and neurons. (b) PC1 of the RMA expression data shows strong correlation with the RNA 5 -3signal bias within each chip. The slope of a chip's RNA digestion curve was used as the measure of 5 -3signal bias. (c) Following a transformation to remove 5 -3signal bias (see text for details), the major source of variation in the data set is now differential gene expression between the two cell types, which are now clearly separable on PC1. (d) PC1 of transformed data is not correlated with the 5 -3signal bias within a chip. (8.23 MB TIF) [file pone.0003964.s003.tif]

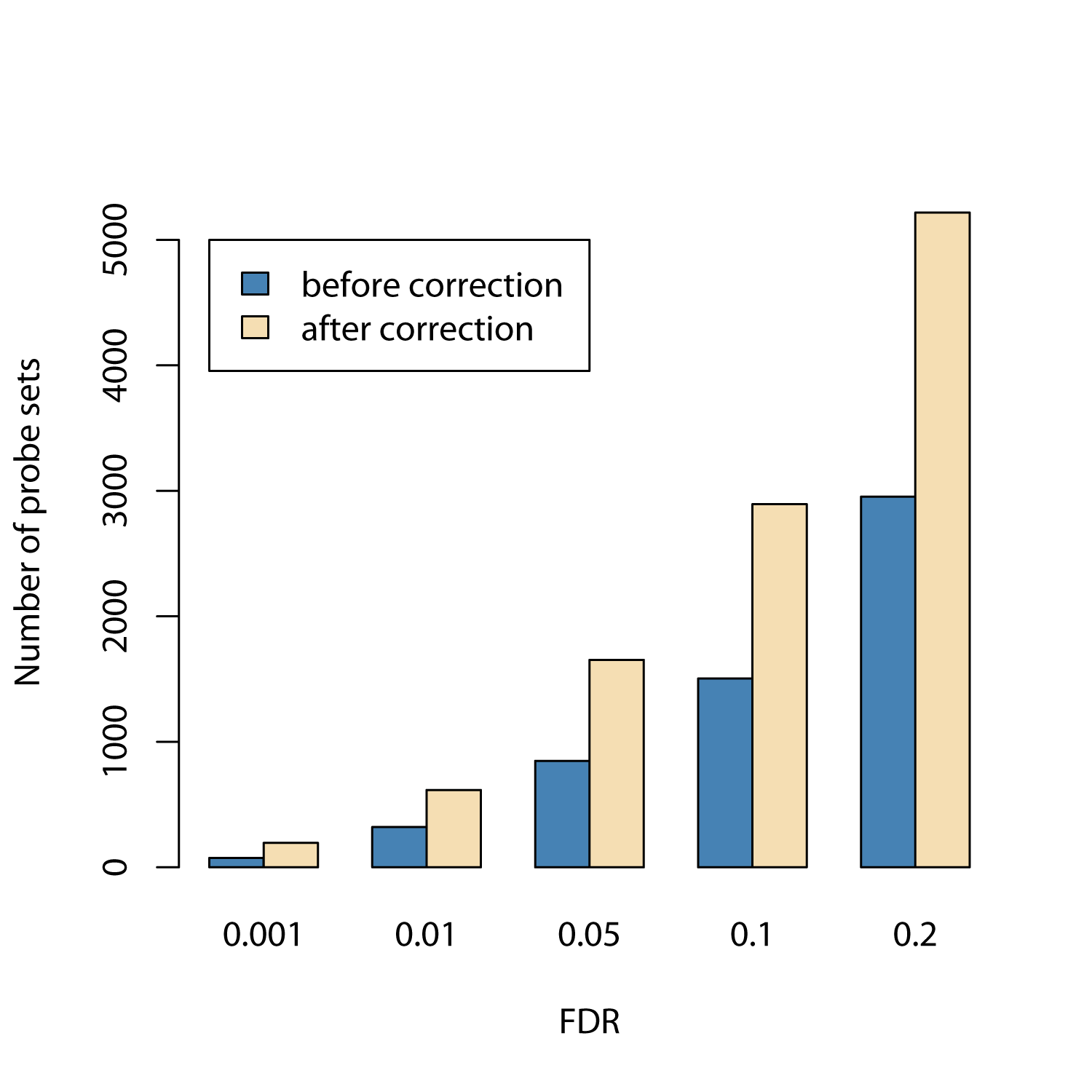

Supplement: Figure S3 — Number of probesets on the Affymetrix GeneChip detecting differential ex- pression between endothelial cells and neurons at a range of false discovery rates (FDR), before and after a correction was applied for 5′-3′ signal bias. For details of systematic bias and correction, see text. (6.77 MB TIF) [file pone.0003964.s004.tif]
